# Resilience Across the Life Course for Women Experiencing Intimate Partner Violence

**DOI:** 10.1177/10778012241236675

**Published:** 2024-03-05

**Authors:** Cara A. Davidson, Christina Safar, Julia Yates, Katie J. Shillington, Nokuzola Nncube, Tara Mantler

**Affiliations:** 1 Health and Rehabilitation Sciences Program, Faculty of Health Sciences, 6221 The University of Western Ontario, London, Ontario, Canada; 2 Department of Psychology, Faculty of Science, Wilfred Laurier University, Waterloo, Ontario, Canada; 3 School of Health Studies, Faculty of Health Sciences, 6221 The University of Western Ontario, London, Ontario, Canada

**Keywords:** life course, resilience, intimate partner violence, age

## Abstract

This study employed a life course perspective to explore the resilience of Canadian women of various ages who had experienced intimate partner violence (IPV). Interpretive description was used to analyze 22 in-depth, semi-structured interview transcripts with women who ranged in age from >19 to 60+ years. Results revealed that developmental age affected service accessibility and effectiveness, historical age shaped abuse normalization, and social age presented barriers and facilitators to women's resilience. This study highlighted the central role of resilience for women of all ages who have experienced IPV and emphasized the need for accessible, effective, and supportive services.

## Introduction

Gender-based violence (GBV) is defined as any harmful act of violence perpetrated against a person because of their gender identity (United Nations Population Fund, 2021). One of the most common forms of GBV is intimate partner violence (IPV), which can be understood as any physical, sexual, and/or emotional abuse in the context of coercive control perpetrated by an intimate partner (Tjaden & Thoennes, 2000). IPV can impact people of all genders, ages, and socioeconomic, educational, and cultural backgrounds (Government of Canada, 2022); however, women are disproportionately affected by IPV. According to police-reported data collected in 2019, of the 107,810 Canadians 15 years and older who reported experiencing IPV, 79% were women and the rates of IPV were 3.5 times higher among women than men (Conroy, 2021). As such, it is not surprising that IPV is a significant public health concern that requires attention from different perspectives. One such perspective proposed by Crann and Barata (2016) suggests that women who have experienced IPV and left the relationship can thrive despite their abusive experiences.

The dynamic process whereby psychosocial and environmental factors interact enabling individuals to grow, survive, and thrive despite exposure to adversity, such as experiences of IPV, is understood as resilience (Howell et al., 2018; Munoz et al., 2017; Prime et al., 2020). Explorations of the interactions of resilience and IPV have largely examined protective individual factors (e.g., self-esteem, positive attitudes, and social support) that enhance resilience in the context of an abusive relationship (Ungar, 2003), or the development of resilience in response to IPV as an adverse experience (Crann & Barata, 2016). For example, Crann and Barata (2016) explored the experiences of resilience among 16 adult female survivors of IPV in Ontario through qualitative, semi-structured interviews. These authors found that women's experiences of resilience were seen through shifts towards resistance, in the experience of control, and ultimately towards positivity (Crann & Barata, 2016), supporting the idea that through the experiences of IPV, women developed their resilience over time. However, these approaches (i.e., protective individual factors or development of resilience in response to adversity) lacks an understanding of *how* environmental factors such as social and cultural contexts influence individual resilience (Ungar, 2003). In a recent qualitative study conducted by Mantler et al. (2022a) of Canadian women (*n* = 14) experiencing IPV, authors found that aspects of a women's environment enabled or created barriers for the women's resilience. These environmental factors included the supports available to women, experiencing IPV during the COVID-19 pandemic, and living in rural communities (Mantler et al., 2022a). Ultimately, these authors concluded that environmental factors must be modified in order to enable women's individual resilience to flourish in the context of IPV (Mantler et al., 2022a).

Resilience in the context of IPV is often examined without attention paid to the age of the women, despite the dynamic nature of resilience. A life course perspective is necessary to encapsulate a more fulsome understanding of how resilience and IPV interact over time and within socio-cultural contexts. Elder and Rockwell (1979) described the life course model as a perspective on human development rooted in the ecological context, specifically the interaction between age, milestones, and time. A life course is understood as the relationship between age and time and can be divided into three subsets: developmental, social, and historical (Elder & Rockwell, 1979). Developmental age marks developmental time as a numeric indicator of age (i.e., getting older every year). Social age is defined as divisions in the life course which create a basis for one's identity by specifying appropriate behaviors based on societal norms (i.e., in Western cultures is it anticipated that someone in their early twenties would be attending post-secondary education; Elder & Rockwell, 1979). Historic age is associated with birth year and relates specific cohorts to the experience of history (i.e., those parenting during the COVID-19 pandemic would view it differently from those parenting in a non-pandemic context; Elder & Rockwell, 1979). Understanding resilience in the context of IPV across the life course requires developmental, historical, and social perspectives on aging to be comprehensive in scope, however, literature has not yet examined this phenomenon. Resultantly, there are extensive gaps in knowledge regarding how resilience interacts with experiences of IPV across time for women at different stages in the life course. A life course perspective is ideally suited to identifying how women experience IPV at different developmental, social, and historical ages. Accordingly, the objective of this work was to explore resilience of women of a range of ages who have experienced IPV.

## Method

This article describes a qualitative sub-analysis from a cross-sectional, mixed-methods project conducted from August 2020 to December 2021. The purpose of this analysis was to explore the resilience of women who have experienced IPV in Ontario across the life course. Ethics approval for the sub-analysis was obtained from the Non-Medical Research Ethics Board in March 2022.

### Study Procedures

To be eligible to participate in the study, participants needed to identify as a woman, have experienced IPV, and have access to a safe electronic device. Recruitment was conducted through advertisements posted to Kijiji, an online community-building and marketplace platform, and Facebook. In addition, women were recruited through posters displayed in women's shelters in Ontario. All recruitment materials invited interested women to email the research team to confirm their eligibility and provide consent to participate.

### Data Collection

All participants (*n *= 22) included in this sub-analysis reported their demographic information and completed a single 60-minute (on average), in-depth, semi-structured interview. Interviews aimed to answer the research question, “How do women of different developmental, historical, and social ages experience resilience in the context of IPV?” The demographic questionnaire included information regarding participants’ age, gender, sexual orientation, indigeneity, level of education, income, marital status, geographic location, and children. All interviews were conducted by one of three graduate research assistants (CD; CF; KS) who were trained by the principal investigator (TM) in safely interviewing women who have experienced IPV. Interviews were conducted via Zoom or the telephone, depending on the participant's preference. Before the interview began, the interviewer established a safety plan with the participant, in the case of an emergency (e.g., being interrupted by the abusive partner). Audio files were transcribed verbatim following the completion of the interviews. Data collection was guided by Guba and Lincoln (1989) and Thorne et al. (1997) principles of auditability, fit, dependence, and transferability. The interview topic guide can be found in Table 1.

**Table 1.** Topic Guide for Semi-Structured Interviews.

Constructs of interest
Perceived life changes during COVID-19 Existing resilience facilitators Barriers to current resilience Facilitators for future resilience Prioritization of resilience strategies

### Data Analysis

**Figure 1.**
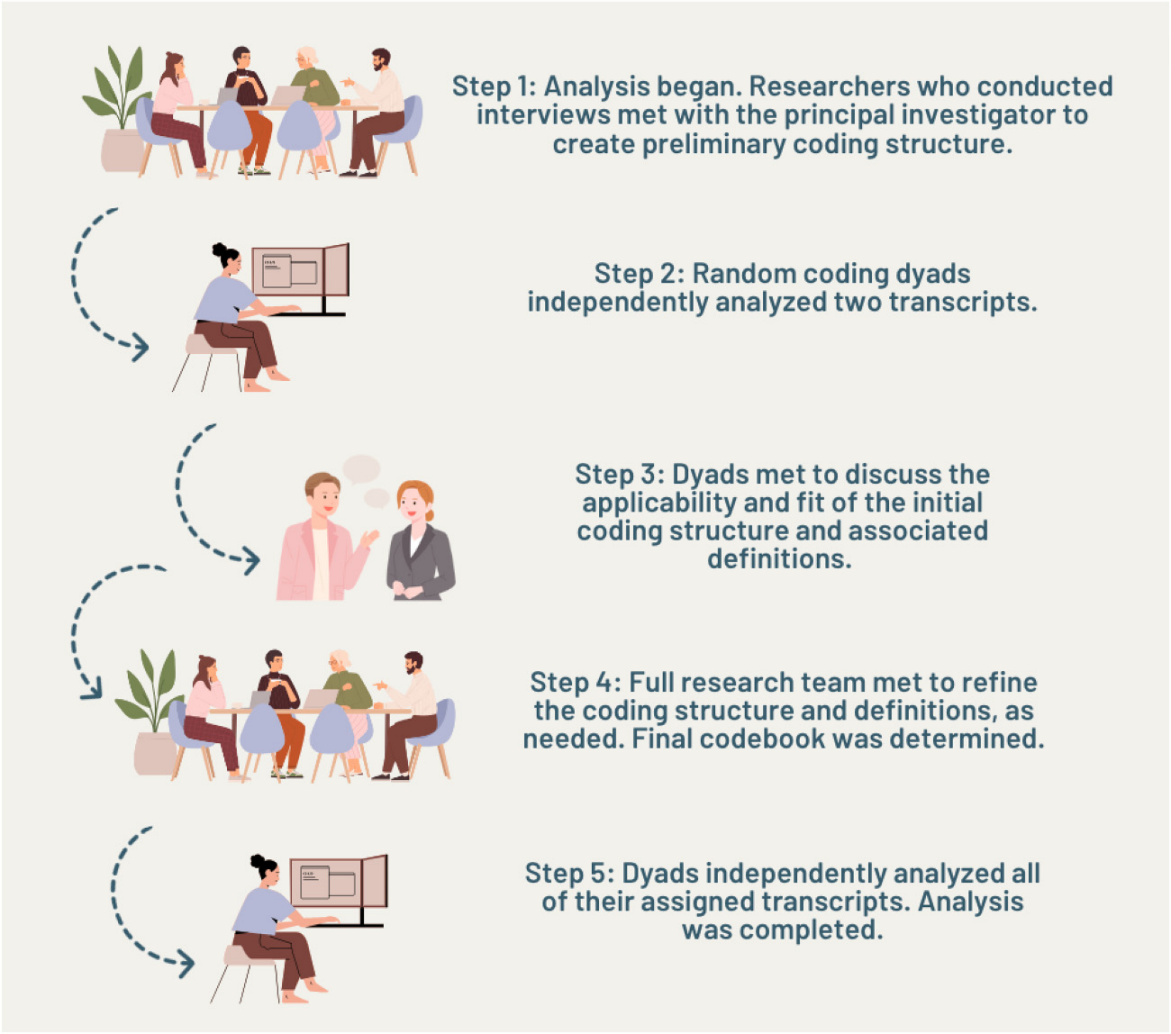
Overview of data analysis procedures.

All authors participated in transcript analysis. Interview transcripts were organized using Quirkos qualitative analysis software (Quirkos 2.4.2, 2021), using Thorne's (2016) interpretive description to guide analysis. Interpretive description “is a grounded approach to articulating patterns and themes emerging in relation to various clinical phenomena” (Thorne et al., 2004, p. 8). Initially, researchers (CD; CF; KS) who conducted interviews met with the principal investigator (TM) to create a preliminary coding structure. The coding structure was developed using the life course perspective (per Elder & Rockwell, 1979) as a guiding theory. Next, random coding dyads, consisting of two authors, were created and each dyad independently analyzed two randomly selected transcripts using open and line-by-line coding (Blundell et al., 2020). Following this, each dyad met individually to discuss the applicability and fit of the initial coding structure and associated definitions to their assigned transcripts. Then, the full research team came together as a larger group to refine the coding structure and definitions, as needed. Through this iterative approach, the final codebook employed the life course perspective as a parent code, with developmental, social, and historical age as child codes. Developmental age was understood as the chronological age of the participant, such that their experience of IPV was rooted in their current age, or their age when they were experiencing IPV. Historical age was defined as norms that were ascribed to people of a specific life stage that affected their experience of IPV. Social age was operationalized as age-related social roles that intersected with their experience of IPV. Quotes were then sorted into age groups (i.e., 19 and under, 20–30, 31–40, 41–50, 51–60, 60+) within each component of the life course perspective. Finally, using the agreed upon codebook, each dyad independently analyzed all of their assigned transcripts (i.e., all transcripts were analyzed at this stage) and utilized memoing to identify theoretical outliers, theorize the relationship and structure of the data, and extract meaning from the data set (Thorne, 2016; see Figure 1 for an overview of the data analysis procedures).

Once analysis was complete, all Quirkos files were merged, and reports were run on codes related to each component of the life course perspective, differentiated by age group.

## Results

In total, 22 women were included in this sub-study with an average age of 41.14 years (*SD* = 13.23 years). Each age cohort (i.e., 20–30, 31–40, 41–50, 51–60) included five participants, while under 19 and above 60 included one participant each due to sampling limitations (i.e., only one participant fit each of these age categories). All participants in this sample identified as a woman (*n* = 22), inclusive of one participant who indicated that they identify as a transgender woman. The sexual identity of most participants was heterosexual (*n* = 14). Overall, this sample had a high education status (i.e., college- or university-level) and low annual household income (i.e., ∼45% < $49,000). Half of this sample (*n *= 11) resided in rural communities (i.e., <30,000 residents). Demographic differences beyond age were not accounted for in this sub-analysis. See Table 2 for full demographic characteristics.

**Table 2.** Demographic Information.

Participant characteristics (*n* = 22)	*n*	%
Age, *M* (*SD*)	41.14 (13.23)	-
Age cohorts		
19 and under	1	4.55
20–30	5	22.73
31–40	5	22.73
41–50	5	22.73
51–60	5	22.73
60+	1	4.55
Gender		
Women	21	95.45
Trans Women	1	4.55
Sexual identity		
Heterosexual	14	63.64
Bisexual	5	22.73
Pansexual	3	13.64
Indigenous		
Yes	1	4.55
No	21	95.45
Educational status		
Some high school	1	4.55
High school	6	27.27
College or university	14	63.64
Advanced degree	1	4.55
Average annual household income		
Less than $20,000	1	4.55
$20,000–$49,999	9	40.91
$50,000–$99,999	5	22.73
Greater than $100,000	4	18.18
Not specified	3	13.64
Marital status		
Single	11	50.00
In a relationship not married, common law, or engaged	2	9.09
Married, common law, engaged	6	27.27
Divorced or separated	3	13.64
Type of community		
Large urban center (100,000 or more)	10	45.45
Urban center (30,000–99,000)	1	4.55
Rural (30,000 or less)	11	50.00
Children		
Yes	9	40.91
No	13	59.09

### Qualitative Findings

Participants described their experiences of IPV in the context of their resilience. Women shared their experiences through direct statements about resilience, for example, “I think I was resilient throughout a lot of the relationship” (N9, 20–30) and “I find that my resilience is being tested more now than during the active assault” (N6, 19 and under). Women also described resilience through indirect terminology, such as “confidence for inner strength” (N1, 20–30), “I had the courage to leave” (JM, 51–60), and “I value myself as a human being” (N11, 41–50). Using a life course perspective, women's direct and indirect descriptions of resilience were analyzed in the context of developmental, historical, and social age as described by Elder and Rockwell (1979). An overview of main themes, sub-themes, and the corresponding age cohorts can be found in Table 3.

**Table 3.** Summary of Themes.

Main theme	Age cohort (s)	Sub-theme
Developmental age	19 and under, 20–30, 31–40, 41–50	Unhelpful services
	20–30, 31–40, 41–50, 51–60, 60+	Inaccessibility of technological services
Historical age	19 and under, 20–30, 41–50, 60+	Normalization
	31–40	Lack of foundations for “normal life”
	51–60	Finance-related
Social age	19 and under	“Non-adult”
	19 and under, 20–30	Friends and family
	31–40, 51–60	Mother
	41–50, 51–60	Wife

#### Developmental

*“I felt like nobody just wanted to talk to me.”—JH (20–30)*

The developmental age of women was described as important to their ability to access effective services related to IPV within all age brackets except for 60+. Experiences of unsupportive health and social services were consistently reported by participants aged 18–50, and experiences of inaccessible services were reported by women aged 20–60.

#### Unsupportive services

Participants across the life course described reaching out to health and social services but having their concerns dismissed by providers. Commonly, participants aged 18–50 experienced unhelpful services across the medical and social service domains. The youngest participant was the only woman who experienced this in an educational setting, as she described trying to advocate for herself and her safety by isolating herself from her abusive partner while at school but being blocked by the school administration. She disclosed:

Once I got him to the vice principal to speak about my issue, she was completely cold, bitter, irritated with me. And I remember that so clearly going in there trembling, like a wet cat. I was scared, and I didn’t know what to do and I was putting my trust in this woman. And she was just irritated and snappy with me… I felt like I was nothing but a burden and I shouldn’t even have asked to make sure those two classes didn’t coincide in that my abuser wouldn't end up in my class. (N6, 19 and under)

This resulted in the participant feeling discouraged avoiding reaching out to educational authority figures again for help. Instead, she demonstrated resilience by choosing to figure out her situation without formal supports.

Experiences of feeling unsupported by services designed to help included crisis lines, medical, and social services for women aged 18–50. Women in the 20–30 and 41–50 age brackets described repeated interactions with crisis lines that were unhelpful. One woman reported, “I tried to call a couple places through my skype but they weren’t really helpful” (JH, 20–30). Another participant stated, “It was, there were three different [crisis] lines I called, and they all hung up” (AF, 41–50). These women demonstrated resilience by repeatedly seeking out assistance; however, they were not met with the support they desired despite their efforts. One participant identified that this was disappointing, by stating “It would have been helpful if a couple of people I reached out to lines, like crisis lines or whatever, were able to at least offer something but I was kind of let down by that” (AF, 41–50). Another participant noted that the experience felt isolating, by stating, “I tried them [phone lines] both more than once and the one always sent me back to the other and I felt like nobody just wanted to talk to me” (JH, 20–30). Overall, women aged 20–30 and 41–50 were unable to meaningfully connect with a crisis counselor despite repeated interactions with crisis lines, which ultimately led to negative perceptions of this service and challenges to their resilience.

Help-seeking from resources that did not produce the desired outcome was also described in the context of medical and social services for women aged 18–30. For example, a participant tried to access local primary care services and found them ill-equipped to support her needs, stating, “… like one little place, like a little doctor's office, once you get into—close to town, they just have like a lot of like, they’re like all, what do you call it, they’re like all the students that are going through to be doctors, so they aren’t particularly that helpful, I mean, really [laughs]” (LM, 20–30). This experience of a lack of helpfulness from a primary care provider was echoed by another participant who described her abuse-related injuries being dismissed by her primary provider. She stated, “My primary care physician undermined some of my serious concerns… I said, I was injured, I believe I had this dislocated, I can't put any weight on it, I can't walk, I think I need assistance moving right now. And he said ‘that’s normal, you’re a woman and girls have this happen a lot. It's just a sprain, it's because girls are more flexible than boys’” (N6, 19 and under). Uniquely, in regard to accessing social services, another participant found accessing a social worker unhelpful because of an extensive waitlist, stating “And then, eventually I’d gotten into a social worker […] and they had tried to set me up in the closest city, and it was a year and a half waiting list” (N9, 20–30).

While many participants reported receiving unsupportive primary medical care and social services, the participant aged 19 and under was the only woman to identify that these experiences were related to her age and the age of her abusive partner, by stating “I feel there's an extreme blind eye to how malicious young people can be” (N6, 19 and under). This ultimately resulted in this participant losing trust in authority as she said, “It just made me want to give up seeing the people that I’m supposed to trust, just not listen to me, you know, thinking I should be able to trust the people, and it turns out, I can’t” (N6, 19 and under). Ultimately, women aged 18–50 reported experiences of help-seeking that did not result in help. Despite their experiences, most women expressed the possibility of reaching out to services in the future, if they were better equipped to support them in meeting their individual needs.

#### Inaccessibility of technological resources

Women who did not reach out to health or social services often described this in the context of services being inaccessible to them. This was noted in the 20–30, 31–40, and 51–60 age groups, where women described technology as a prominent barrier to resource accessibility. However, the reasons for inaccessibility varied between age groups: women aged 51–60 identified a lack of knowledge of technologically based services as a barrier, while women aged 20–40 described extensive knowledge of privacy concerns associated with digital service use. For example, a participant aged 51–60 stated “I was more aware of in person services. I didn't know that they were conducting things online. I had no idea” (JM, 51–60). Another participant echoed this sentiment, saying that “I didn't know if they had these or not, but there should be support groups… But with the pandemic, I really wasn't able to access anything [in-person]” (FJ, 51–60). This suggested that older women who were not tech-savvy found it difficult to find and/or access supports online.

Participants aged 20–40 demonstrated expertise in navigating privacy concerns associated with technology use in the context of IPV. For example, one participant identified privacy concerns associated with using phone or email to access services by sharing that call logs are not private and can endanger women, “You call someone it's going to show up on your bill. You know, you might be able to delete it from your call log. But the actual paper phone bill, it's going to show up there. And you know, the question, well, ‘who did you call and you talked for 10 minutes’, you know, like that. You can’t hide that stuff” (AL, 31–40). Another woman spoke to privacy concerns associated with emailing her therapist for support by stating, “Like we would sometimes email each other, but I couldn’t really go into depth with how I was doing” (LM, 20–30). Overall, technology-based services were positioned as potentially inaccessible to women experiencing IPV, however, the reasoning varied by age. Women aged 51–60 reported difficulty with virtual services due to knowledge barriers, while women aged 20–40 reported privacy concerns associated with technology.

#### Historical

*I found it extremely hard to—to find that normal life and to know what that was, or what that was supposed to be like.— N5 (31–40)*

Experiences of IPV were rationalized by participants according to various age-related norms in each age bracket. These rationalizations included the normalization of IPV (19 and under, 20–30, 41–50, 60+), a lack of foundations for “normal life” (31–40), and finance-related reasons (51–60). A common experience across participants was a general lack of attention to their individual resilience stemming from the “normalcy” surrounding their experiences of IPV.

#### Normalization

Many participants echoed the feeling of their experiences of IPV being normalized based on societal norms. Participants between the ages of 18 and 30 shared feelings of being too young at the time they were experiencing violence to truly recognize it as violence. One participant described that “I was too young to really even put two and two together. I kind of figured it was just normal” (N6, 19 and under). Moreover, many within this range believed the violent moments within their relationships did not equate to abuse but rather, were normal occurrences within all relationships. One participant explained the oscillation in violent experiences, stating “It didn’t happen all the time. I mean, we fight, but then things would get better again, and things are okay for a few weeks, or sometimes even a few months at a time” (N1, 20–30). The idea that their experiences of violence were one-off situations was echoed by participants, who noted this as a barrier to their resilience in being able to survive on their own. A participant explained this roadblock, “At that time I didn’t have anywhere else to go in terms of a place to live as well. So [I thought] ‘okay, I’ll just kinda continue this for a while and see how things go’” (N1, 20–30). Another participant echoed the experiences of staying due to normalizing these seemingly few and far between experiences of abuse by providing a metaphor of the slow simmer of abuse:

I feel like it kinda slowly progresses… if you put a frog in boiling water, then it’ll hop out, but if you…put the frog in a pot of water and then start boiling, it’ll stay there, so kind of putting that metaphor in real life experiences like, you kinda do it when you’re like okay, like that was just the one time, that like, they hit me, and then it was well they said this, but then they apologized. (N9, 20–30)

On the other hand, participants in the 41–50 age group rationalized their experiences of abuse by excusing their partners’ abusive behaviors. One participant acknowledged that she did not allow herself to believe that “we were a couple that was not normal” (N11, 41–50) and instead, chose not to face the reality that what she was experiencing was, in fact, abuse from her partner. The rationalization of these behaviors may have posed a barrier to participants’ resilience via feelings of self-doubt and questioning their experiences.

Conversely, for the 60+ age group, feelings of normalcy surrounding experiences of IPV stemmed from their lack of focus on the violence. For one participant, this resulted in a lack of attention to their individual resilience given the belief they had nothing to “bounce back” from. This participant explained “To be honest, I don't take any action… I just ignore” (AE, 60+). This participant stressed the importance of shifting their focus from their experiences of IPV to other, more controllable, actions that could help them cope with day-to-day life including physical acts of self-care. For example, the participant described, “Once a week I bake something new” (AE, 60+).

#### Lack of foundations for “normal life.”

For those participants in the 31–40 age range, societal norms around what a “normal adulthood” was comprised of posed a barrier to realizing the impacts or presence of IPV experiences. One participant described this by saying, “I never really got to experience what a normal life was, before him…” (N5, 31–40). Getting into abusive relationships at a young age was noted as a key factor for women later in life due to a lack of knowledge surrounding what “healthy” relationships entailed. Moreover, lacking society-driven, age-appropriate experiences served as an additional barrier to women cultivating their resilience following experiences of violence, given their lack of knowledge surrounding what normal looked like at that point in their lives. One participant explained that “[she] never really had much of a chance at having a normal adulthood, because [she] was in a relationship at a very early age” (N5, 31–40).

#### Finance-related

In many relationships, an increase in the historical age of both partners is accompanied by the integration of finances. When this occurs in the context of IPV, women may feel as though they must remain in their current relationship due to being unable to financially survive on their own. One woman explained that since “[they] bought a home together” (RB, 51–60) she couldn’t leave her situation easily. Moreover, experiencing IPV for a prolonged period of time may reduce or prevent a woman from being financially self-sufficient. One woman aged 51–60 provided the example of not having enough money on her own to make another down payment, causing her to conclude that “[she] just can’t walk away” (RB, 51–60).

#### Social

*This is something very basic, you know, you find a life partner, you build a life together, you succeed, you grow together, you grow old together, you get- well, you get married.— AM (51–60)*

The importance of social roles for women in the context of IPV was noted by participants in every age bracket (19 and under to 60+). The social roles described by women included being a “non-adult” (19 and under), friend (20–30), daughter (19 and under and 20–30), mother (31–40, 51–60), wife (41–50, 51–60), and caregiver (60+). Participants described their social roles as both helping (friend, daughter, sister and mother) and hindering (non-adult, wife, mother, and caregiver) their resilience both within and after an abusive relationship.

#### Non-adult

The non-adult role was prominent within the 19 years and under bracket, where the participant described a desire to achieve traditionally adult social roles such as employment, education, and relationships. The individual described working to achieve these roles by saying, “Before or right after my assault, I couldn't have a job because I was so sick, and now, yeah, it's a minimum wage job, but I have a job. I'm back in school, I have a partner, I am in counseling. Those are huge steps for me” (N6, 19 and under).

The importance of adult roles was rooted in a desire for independence, as she described, “I felt that my independence was stunted after my assault and my physical disability contributed to that” (N6, 19 and under). Independence was closely tied to personal resilience for this participant, as she reported that “[My] feeling of not being able to be independent, damaged my resilience so much” (N6, 19 and under). Once this participant was able to achieve adult social roles and benefit from their associated social and economic capital, she described a positive change in her mental health, “As soon as I got that independence, I was like, wow, I have come so far, I am becoming an independent person. I'm really, being able to fill that role of an adult, which made me happy, it made me happy to be able to do that. It made me feel like I was doing good, and it made me feel like I could keep going” (N6, 19 and under).

#### Friends and family

Reliance on friends and family, as a friend, sister, and daughter, was described as essential to exiting the relationship by participants in the 21–30 age bracket, but detrimental in the 19 and under age bracket. For one participant, her father's advice when she was growing up was formative for her realizing that her partner was abusive, as she described:

I think I was resilient throughout a lot of the relationship, in a way, just I don't know, my dad was—always told me, I don't know he always talked in metaphors when I was growing up, and he'd be like you know how you break a horse's spirit by basically just beating them down and then—and then they'll listen to your commands eventually, that's how you tame a wild horse, and so he kind of, I don't know, it just really stuck in my head the entire time… (LM, 21–30)

Two participants, who identified their social roles as a sister and a daughter, respectively, identified the importance of drawing on these roles for support during the process of exiting the relationship. One participant expressed that a friend helped to mitigate her fears of leaving by assisting with the logistical process, stating that, “But my friend helped me in terms of kind of arranging the logistics, like moving this stuff to the car and then making sure that there's enough time buffer, so he wouldn't walk in while we were kind of packing and everything” (N1, 21–30).

After leaving, one woman described that her sister helped her to prevent contact after leaving; she said, “And we just blocked him on all social media and just like, changed the key, like the lock on our door, because…he had a spare key” (N9, 21–30). However, the role as a daughter was described as a barrier to resilience by the youngest participant, who stated, “I was a burden on my parents and my family, and everybody for not having that income, not being independent” (N6, 19 and under) after exiting her abusive relationship. It appeared that it was preferable to draw upon social resources for time and support, but not income, to support one's resilience among these participants.

#### Mother

Several women in the 31–40 and 51–60 age brackets who were mothers described the motherhood social role as a source of strength to exit their abusive relationship and to keep going after leaving. However, the 60+ participant identified motherhood as a challenge to leaving her relationship.

Women aged 31–40 first recognized that the abusive relationship was problematic for their children, and subsequently drew upon their motherhood role as motivation to leave the relationship. One participant described this by stating, “Definitely the motivation of- of wanting to do the best thing for my daughter, regardless of what that may be, or regardless of how hard that may be. Wanting to give her the best life possible has definitely been a game changer” (N14, 31–40).

Another mother emphasized how her role as a mother promoted her resilience when trying to get her son out of an abusive home by stating:

My son is a bit older, he's a preteen. So he knew a lot of what was going on and he he's, you know, experienced some of the same psychological abuse. So having things like the cameras around all the time watching us and recording us, it was really hard because I would sometimes try to pull him aside and just say, like, you know, it's, it's okay, like, I'll find a way I'll find the way… (AL, 31–40)

Similarly, women aged 31–40 and 51–60 described their role as a mother as a source of strength to keep going both within and outside of the relationship. One participant identified that having kids in the context of an abusive relationship improved her resilience, stating, “But when you have kids too, sometimes something pulls you up saying you gotta keep on going” (N12, 51–60). Another participant echoed this sentiment in the context of exiting the relationship, sharing, “Honestly, I would have to say that I didn’t have a whole lot of [inner strength], after the first relationship. I—I really took a real bad turn, after that relationship ended, I think that most of my inner strength didn’t start to really show, and I didn’t really start to feel it until after I left my son's father, and it was my son who gave me that strength” (N5, 31–40).

Uniquely, the participant aged 60+ described that she struggled to exit her relationship because of her children expressing that they want her to stay with her partner. She described that her children did not know the extent of the abuse, stating, “Because everything is closed door, so they don't see what's going on you know” (AE, 60+). Resultantly, her children pled with her to stay with their father that complicated her ability to leave, as she described, “You know my kids say, you know, my dad is not bad, you can handle him, you can handle him. Sometimes I feel like I'm gonna give up you know? I’m tired, I’m 67 years old, I’m tired, my life, I’m just tired. And they say mom, you know, because they, they don’t see everything” (AE, 60+).

When describing motherhood as a social role, women were divided in terms of it being either a source of motivation and strength or an additional challenge when exiting an abusive relationship and living after experiencing abuse.

#### Wife

Two participants in the 41–50 and 51–60 age groups identified that their role as a wife impacted their experiences of IPV and resilience. Being a wife was described as a social mold that the women aspired to fit but struggled to achieve because it asked them to tolerate the abuse they experienced. For example, one woman shared, “I was trying to- to be the best wife possible, to fulfill my- my- my duties, my responsibilities, to be faithful, to keep a house clean, to bring in some money, to make sure to pay the bills and all that” (N11, 41–50). This participant worked to fulfill the wife social role and felt as though she was the problem for being unable to do so, stating, “I was perceiving this as I was doing something wrong, as an isolated situation, nobody else is going through this” (N11, 41–50). This feeling of doing something wrong was heightened by the fact that her peers who were wives did not appear to have relationship problems, as she described, “But maybe if their [her friends] relationships weren't going too well, then maybe that would have helped me to be more open and honest with them from the get-go, and maybe it would have encouraged me to leave my husband” (N11, 41–50). Similarly, another participant described how her understanding of being a wife dissuaded her from leaving, stating, “I was against at the time divorce at all costs, so you know raised by a grandmother, old traditional values, you know, you're a virgin when you get married, and then you stay together no matter what, so all that weighed heavily on me, and both probably prevented me from taking action you know, quicker, sooner than later of course” (AM, 51–60). Both participants who described being a wife as a social role described how they experienced conflict when deciding whether to leave their abusive partner because of social expectations implying that they should stay.

## Discussion

The purpose of this study was to explore resilience using a life course perspective among women who have experienced IPV in Ontario. Developmentally, women revealed chronological age was important in accessing services with women across all age cohorts identifying unsupportive services and inaccessibility of technology-based services as barriers to resilience building. Historically, experiences of abuse were rationalized using age-related norms with 18- to 30-year-old participants having difficulty identifying they were in an abusive relationship, 40- to 50-year-old participants excusing the abuse due to life circumstances, and 60+-year-old participants viewing abuse as a normal within intimate relationships. These age-related norms created the foundation where individual resilience was undermined. Socially, the roles women held such as friend, daughter, and sister bolstered resilience, where non-adult, wife and caregiver undermined resilience. The role of mothering both bolstered and undermined resilience. Using the three subsets of the life course perspective, that is, developmental, historical, and social age, clarified how age and time interreacted and shaped resilience among women who have experienced IPV.

Women across all age cohorts described the negative impact of unsupportive services as undermining their resilience. While resilience has largely been understood as a personal resource, recent research has underscored the influence of environmental contexts and having access to resources as foundational in supporting the resilience of women, including women experiencing IPV (Mantler et al., 2022b). The impact of unsupportive services, whether it be services that do not meet women's needs or are not offered in ways that are supportive to women, have been established as a long-standing barrier to service use for women experiencing IPV (Ford-Gilboe et al., 2015). In a community sample of 309 Canadian women who had experienced violence, it was found they had service use rates ranging from 2–92× that of the general population, underscoring that services were not meeting the needs of women. Understanding why services are not meeting the needs of women experiencing violence has been further nuanced in a recent systematic review by Robinson et al. (2021), which reported that the main barriers to help-seeking were system failures, such as interacting with a service not sensitive to experiences of violence. Women experiencing violence need access to affirming services, that is, services that understand how violence impacts the lives of women and support women experiencing the consequences of violence to build resilience.

Technological barriers were identified as impacting not only access to services but also undermining resilience. While technological barriers have been well documented among older cohorts (Emezue, 2020), novel to this study was the presence of these barriers for all women. Older cohorts described lack of knowledge whereas younger cohorts described privacy concerns as the technological barrier. While many technology-based safety strategies have been identified as useful for minimizing the risk of women experiencing violence, such as safe internet browsing and clearing phone and internet histories (Emezue, 2020), this study underscored that these well known safety strategies do not address the privacy concerns of younger cohorts of women. This finding is consistent with a study by Moreno et al. (2014) that found younger women tend to be skeptical of existing safety measures in social media technologies, but unique to this study was that this privacy concern extended beyond social media to include accessing any services through technology. Taken together, there is a need for technology-based IPV services that ensure safety and privacy concerns are clearly, fulsomely addressed so that women feel as comfortable as possible when engaging with services.

The influence of prevailing societal norms on violence has been established in the literature, particularly in relation to older cohorts. Societal norms dictate acceptable roles and behaviors of different generations (Blackstone, 2003). Older women would have been raised in a time when society placed value on women being homemakers and caretakers (Sprott et al., 2003), these roles were submissive in the family context which would reinforce the idea that IPV be kept as a family matter (Band-Winterstein, 2015). IPV perpetrated in old age is often regarded less seriously. In addition, more sympathy tends to be extended to the perpetrators because of their perceived physical frailty or infirmity, which complicates the ability to estimate the true prevalence and consequences of IPV in older individuals (Stewart et al., 2021). However, unique to this research was the realization that 18- to 30-year-old women often did not recognize the signs of being in an abusive relationship. These cohorts described needing to come to the realization that they were in fact in an abusive relationship and having to grapple with this prior to being in a position to deal with the abuse. Moreover, 40- to 50-year-old women often excused the abuse by examining how environmental stressors were the cause of violence. This rationalization appears to be a remanent of the acceptance of abuse from older cohorts. While there is evidence that much work has been done to stop the normalization of GBV (Adelman et al., 2016; Choo et al., 2019; Wexler et al., 2019), there is still much work to do. Specifically, it is important to ensure that the public is educated on what constitutes a violent relationship, such that women can identify if they are in these situations sooner and hopefully seek help as needed.

The social roles women hold in society often overlap and have been linked to resilience (Hirani et al., 2016). A commentary by Smyth and Sweetman (2015) described the connection between roles women hold, their contributions to society, and whether this bolsters or undermines resilience. Women in this study described being a friend, daughter, and sister as motivation to bolster resilience. They described being seen as a non-adult, wife, and caregiver as key factors in undermining their resilience. This sense of duty associated with being a wife and/or caregiver role has been established a reason for staying in abusive relationships in past research studies (Band-Winterstein, 2015) while other mothers had their resilience undermined because they felt they needed to stay in the relationship because of the children (Shillington et al., 2022). This study underscored the varied and overlapping social roles women hold and how they shape resilience. There is a need for IPV responses to be sensitive to the many roles women hold, particularly in regard to how these roles are intersecting with help-seeking behaviors, or the ability to leave abusive relationships.

## Limitations

Some limitations should be considered alongside the results of this work to contextualize the findings. Due to this study being a sub-analysis of pre-existing interview transcripts, participants were not directly asked how their experiences of developmental, social, and historical age contributed to their resilience in the context of IPV. Thus, these results may not fully encapsulate women's perceived interactions between age and IPV throughout the life course. It is recommended that future IPV-related research include exploring the life course as a primary objective to validate these findings. Additionally, only one participant was included within each of the youngest and oldest age brackets due to limitations in sampling. As such, the themes reported for these groups may not be generalizable to other women of the same age. Future research should deliberately oversample teenage and older adult participants to address their underrepresentation in IPV-related life course research. Moreover, the developmental perspective did not include the 60+ participant because her transcript did not appear to address this perspective. Resultantly, the relationship between resilience and IPV for women over the age of 60 using a developmental perspective cannot be determined from these results. An additional limitation is that the full extent of the life course was not represented in this work. It is known that women of any age can experience IPV, but our sample was limited to those aged 18–67. Of particular absence are experiences of dating violence for teenagers and elder abuse for older women; future research should consider adopting a life course perspective in the context of these age-related forms of IPV.

## Conclusion

IPV is a prevalent public health concern in Canada that affects women of all ages. Experiences of IPV vary across women of different ages when viewed through a life course perspective. At the time of writing, this study is one of the first to use a life course perspective, inclusive of developmental, historical, and social ages, to examine Canadian women's experiences of resilience in the context of IPV. The findings of this study suggested that developmental age was central to women's ability to access effective services related to IPV (ages 18–50); specifically, women across the life course reported being affected by unsupportive social services (ages 18–50) and experiencing accessibility and privacy concerns with technology-based services (ages 20–30, 31–40, and 51–60). Historical age was identified as central to women's conceptualization of healthy and “normal” relationships based on social norms across the life course (all ages), which impeded their ability to recognize their partner's behaviors as abusive. The social age perspective elucidated how women's social roles could act as facilitators (friend: ages 20–30, daughter: ages 19–30, and sister: ages 21–30) or barriers (non-adult: ages 19 and under, wife: ages 40–60, and mother: ages 30–40 and 50–60) to resilience in the context of IPV. There remains a need for supportive, effective services for women experiencing violence to build their resilience. Moreover, technology-based services must ensure safety and privacy concerns are addressed to promote comfort and trust for women who use these services. Given the importance of social norms to the experiences of IPV as described by women, it is necessary that the public is educated about recognizing IPV and how to safely seek help. Services must be responsive to the social roles that women hold and how they intersect with help-seeking and leaving the relationship. By improving IPV-related service accessibility and effectiveness and increasing public awareness and understanding of IPV, women across the life course can be empowered to recognize and safely exit abusive relationships.
